# Image reconstruction and elongation artifact reduction for a dual‐panel dedicated prostate PET scanner

**DOI:** 10.1002/mp.70298

**Published:** 2026-01-27

**Authors:** Abdollah Saberi Manesh, Mehdi Amini, Yazdan Salimi, Katayoun Doroud, Crispin Williams, Themistoklis Williams, Hossein Arabi, Habib Zaidi

**Affiliations:** ^1^ Division of Nuclear Medicine and Molecular Imaging Geneva University Hospital Geneva Switzerland; ^2^ Picotech St Genis Pouilly France; ^3^ Department of Nuclear Medicine and Molecular Imaging University of Groningen, University Medical Center Groningen The Netherlands; ^4^ Department of Nuclear Medicine University of Southern Denmark Odense Denmark; ^5^ University Research and Innovation Center Óbuda University Budapest Hungary

**Keywords:** artifact, deep‐learning, dual‐panel, organ‐specific PET, prostate, reconstruction

## Abstract

**Background:**

The development of PET scanners dedicated to high temporal and spatial resolution organ‐specific imaging is an active research area, motivated by the need for cost reduction, improved lesion detectability and quantification in specific clinical scenarios, as well as by ongoing hardware and software innovations.

**Purpose:**

This study investigates and compares various image reconstruction strategies for a dual‐panel prostate‐dedicated PET scanner (ProVision), which features four‐layered dual‐readout time‐of‐flight depth‐of‐interaction detectors and a 22‐position acquisition protocol to improve angular coverage.

**Materials and methods:**

A list‐mode MLEM algorithm with multi‐ray modeling was developed and optimized using a scaled NEMA image quality phantom to determine optimal number of rays and iterations. These parameters were then used to reconstruct data from both simulation and experimental acquisitions, including an anthropomorphic pelvis phantom, named Adam‐PETer. Four reconstruction approaches were evaluated: classical MLEM; MLEM with embedded shift‐variant point spread function (PSF) modeling; a hybrid list‐mode reconstruction; and a Swin‐UNETR‐based deep learning model applied as a post‐reconstruction enhancement to MLEM images. Performance was assessed using contrast recovery coefficient (CRC), contrast‐to‐noise ratio (CNR), and contrast‐to‐noise consistency (CNC), on both a scaled NEMA phantom and an experimental anthropomorphic phantom.

**Results:**

In the scaled NEMA phantom simulation, the Swin‐based method yielded the highest CNR and CNC, especially for the smallest spheres, thereby outperforming both standard MLEM and the hybrid algorithm. In the Adam‐PETer experimental prostate phantom, the CNR was 10.43 for MLEM, 14.48 for Hybrid, and 13.85 for Swin for the larger lesion (10 mm). The CNR values were 2.28, 3.03, and 4.35, respectively, for the smaller lesion (8 mm). CNC values also varied across methods, with Swin achieving the best result for the smaller lesion. These findings indicate that model‐based and learned methods offer complementary strengths depending on lesion contrast and size.

**Conclusion:**

The PET scanner‐adapted reconstruction combined with deep learning refinement improves image quality in dedicated, limited‐angle PET systems.

## 1 INTRODUCTION

Prostate cancer is one of the most frequently diagnosed malignancies in men and remains a significant cause of cancer‐related mortality worldwide.^1^ Molecular imaging using PET has become an essential tool in detecting, staging, and monitoring this disease, particularly when combined with prostate‐specific molecular imaging probes.^2^ To improve the accuracy and resolution of prostate‐targeted imaging, dedicated PET systems have been developed with scanner geometries and acquisition protocols targeted to improve resolution and sensitivity within the pelvic area.^3^ These systems are designed to maximize detector proximity, angular sampling, and spatial resolution, thereby enhancing lesion visibility and quantitative accuracy in the field‐of‐view.^4^ Their compact size also enables high‐sensitivity acquisition (owing to the proximity of detectors to the patients) leading to lower radiation doses and shorter acquisition times, features beneficial for both patient comfort and clinical throughput. Expanding on this trend, non‐cylindrical and reconfigurable PET scanner designs have been explored to further enhance application‐specific imaging performance. For instance, the Active‐PET scanner employs an adjustable gantry size and DOI–TOF detectors that combine depth‐of‐interaction (DOI) and time‐of‐flight (TOF) measurement capabilities to optimize spatial resolution and sensitivity across multiple anatomical sites, including the prostate.^5^ These advances reflect a shift toward more flexible, task‐driven PET configurations.

A practical and increasingly adopted approach consists in using dual‐panel PET systems, where two opposing flat detectors are positioned around the target organ. While these systems offer enhanced proximity, lower cost, and easier integration compared to full‐ring scanners, they inherently suffer from limited‐angle data acquisition, which leads to incomplete projection coverage and characteristic artefacts, most notably the elongation artefact caused by insufficient angular sampling.^6^, ^7^, ^8^ While dual‐panel PET scanners are cost‐effective and easier to design and manufacture compared to full‐ring systems, these image artefacts must be effectively addressed to generate clinically valid images. In this regard, significant efforts have been made to develop dedicated image reconstruction algorithms, post‐reconstruction elongation correction methods, and deep learning‐based solutions. Specifically, techniques incorporating regularization components, resolution recovery or deconvolution methods, and deep learning models for artefact correction have all been reported to mitigate the inherent limitations of dual‐panel PET imaging. Even small panel misalignments can introduce inconsistencies in line‐of‐response definition, leading to spatial blurring and bias in quantitative uptake estimates. Recent calibration and flexible‐geometry studies highlight the need for reproducible positioning schemes and validation metrics in non‐cylindrical PET systems, which motivates the standardized 22‐step protocol adopted here.^9^, ^10^, ^11^, ^12^, ^13^, ^14^, ^15^, ^16^, ^17^

In addition to algorithmic advances, recent hardware developments have achieved remarkable improvements in coincidence‐timing resolution (CTR), approaching or even surpassing the 100 ps threshold. For example, multi‐anode micro‐channel‐plate photomultipliers have demonstrated 50 ps CTR,^18^ double‐ended fibre‐readout designs have reached 98 ± 1 ps CTR,^19^ ASIC‐based front‐end electronics, such as FastIC+ and multiplexed LVDS readouts, have achieved ∼100 ps timing in compact detector configurations.^20^, ^21^ These advances, together with theoretical and simulation analyses exploring 10–50 ps timing regimes,^22^, ^23^ illustrate the strong research momentum toward next‐generation TOF‐PET detectors. Continued progress in this direction is expected to further mitigate elongation artefacts in limited‐angle dual‐panel geometries by improving event‐localization accuracy.

The ProVision PET scanner is a prostate‐dedicated dual‐panel system equipped with dual‐side readout (i.e., readout at both ends of the scintillation crystal to estimate depth‐of‐interaction) DOI and TOF enabled detectors and a motorized mechanism to perform acquisitions across 22 discrete panel positions. This repositioning strategy partially compensates for angular under‐sampling by improving spatial coverage in both axial (*Z*) and transaxial (*X*) directions. Previous studies on ProVision have demonstrated feasibility in simulation and phantom experiments, establishing a strong foundation for algorithmic development.^24^, ^25^, ^26^ In earlier work, we introduced shift‐variant PSF modeling into the reconstruction process, significantly improving spatial resolution and reducing elongation artefacts.^8^ In this study, we extend this framework by proposing a hybrid image reconstruction pipeline that incorporates list‐mode multi‐ray MLEM, resolution modeling, and post‐reconstruction enhancement using a Swin‐UNETR‐based deep learning network trained on both clinical and synthetic 3D data.

This work is aligned with recent advancements in PET image reconstruction^27^, ^28^, ^29^ and draws upon hybrid optimization strategies, such as hybrid reconstructions with total variation (TV) regularization and benefiting from deep‐learning‐based solutions.^11^ Our evaluation leverages both Monte Carlo simulated and experimental studies to assess image quality in terms of contrast recovery, noise suppression, and lesion detectability. The combined results illustrate the value of integrating physical system modeling with learning‐based resolution recovery for next‐generation prostate‐dedicated PET imaging.

## 2 MATERIALS AND METHODS

### 2.1 PET system design & geometry

**FIGURE 1:**
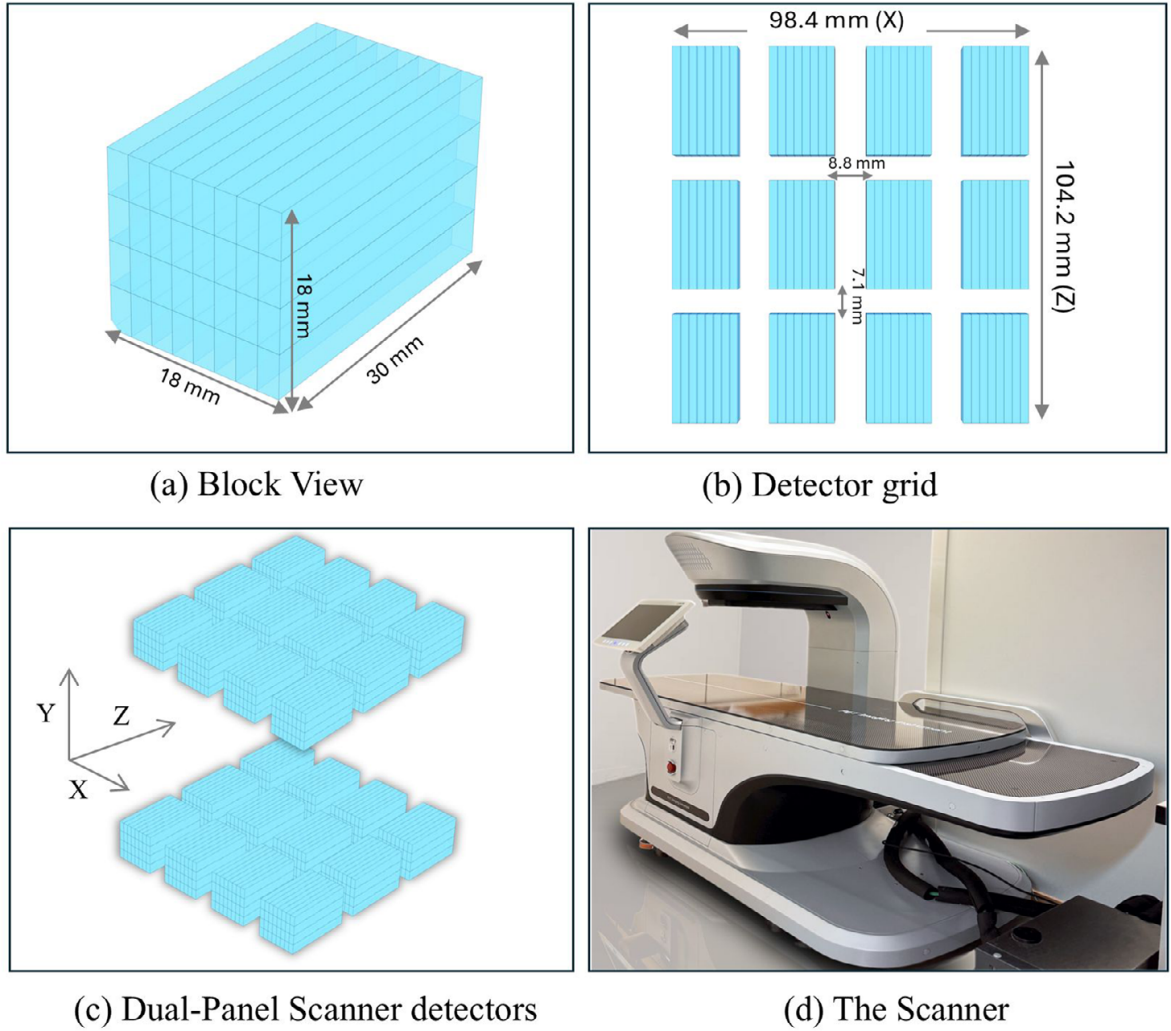
Geometry and design of the ProVision dual‐panel PET scanner. (a) Detector block composed of stacked LFS crystals (18 × 18 × 30 mm^3^). (b) Arrangement of 3 × 4 detector modules per panel, covering 98.4 mm (X) × 104.2 mm (Z). (c) Dual‐panel scanner configuration with matched X–Y–Z orientation. (d) Photograph of the assembled ProVision scanner. LFS, lutetium fine silicate.

The Provision scanner employs a dual‐panel configuration specifically designed for prostate‐dedicated PET imaging. Each panel consists of a 3 × 4 grid of detector modules, covering a total area corresponding to 98.4 mm along the *X*‐direction (right to left) and 104.2 mm along the *Z*‐direction (caudal to cranial). As illustrated in Figure 1, each detector module comprises a 3D array of 8 × 1 × 4 lutetium fine silicate (LFS) crystals, with individual crystal dimensions of 2.25 mm along the *X*‐direction and 30 mm in depth. A DOI scheme is implemented using four stacked crystal layers, each 4.5 mm thick, providing an interaction‐depth resolution of approximately 4.5 mm FWHM and improved spatial localization along the crystal length. Crystals are read out from both ends using strip‐shaped silicon photomultipliers (SiPMs), providing an energy resolution of ∼14% FWHM at 511 keV. An independent point‐irradiation measurement of the detector modules confirmed that the axial (*Z*‐direction) spatial resolution is approximately 3.5 mm.^26^ Detector modules are spaced with 8.8 mm gaps along the *X*‐direction and 7.1 mm along the *Z*‐direction to accommodate electronics and thermal management. Each detector layer consists of eight LFS crystals coupled optically to eight strip SiPMs positioned at opposite ends of the array (four per side). This detector design allows every SiPM to collect light from all crystals within the layer while each crystal is being read by all the eight strip SiPMs. The complete system comprises two opposing planar panels arranged along the *Y*‐direction (anterior to posterior), forming a compact, limited‐angle geometry optimized for high‐resolution prostate imaging and supporting TOF capabilities with ∼180 ps coincidence timing resolution.^25^, ^26^

### 2.2 Detector positioning and acquisition protocol

The ProVision system employs a dual‐panel PET configuration in which the bottom detector panel is fixed beneath the patient bed, while the top panel is mounted on a vertically adjustable gantry. Both panels are equipped with motorized actuators that allow movement along the *X*‐direction, with a motion range of ± 104 mm. The bottom detector also has a movement range of ± 45 mm in the *Z*‐direction, which in combination with the bed movement can simulate relative movement of ± 22.5 mm with the top panel in the *Z*‐direction. Panel motions are software‐controlled and automatically verified before each acquisition, providing reproducibility within ± 0.5 mm.

The 22‐step acquisition protocol represents the scanner's predefined sampling scheme developed jointly with the system manufacturer to enhance angular coverage and mitigate elongation artefacts. These positions were optimized during system development to cover symmetric, offset, and tilt configurations that stress‐test geometric alignment and sampling uniformity across the prostate field of view while remaining within mechanical limits (± 104 mm *X*, ± 22.5 mm *Z*). This work focuses on reconstruction methodology; scanner motion control and hardware configuration follow the system's predefined specifications.

**FIGURE 2:**
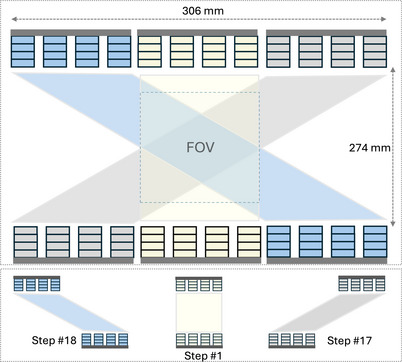
Representative dual‐panel configurations of the Provision PET system illustrating the 22‐step acquisition protocol. Three example positions (steps 1, 17, and 18) are shown relative to the field of view (FOV). The complete coordinate list is given in Table S1.

During all acquisitions taken for this study, the inter‐panel distance along the *Y*‐direction was fixed at 274 mm to maintain a constant gap between the opposing panels. A total of 22 positioning steps were defined, each corresponding to a unique combination of top and bottom panel coordinates. These positions were predefined to ensure diverse angular projections and improve overall sampling uniformity. Figure 2 illustrates three representative configurations (steps 1, 17, and 18) of the 22‐step acquisition protocol, showing the range of dual‐panel motion relative to the field of view (FOV). The complete coordinate list for all 22 positions is provided in Table S1. In positioning step #1, both panels are centered at the origin (0, 0) in the *X*‐ and *Z*‐directions. In step #17, the top panel is shifted to +104 mm and the bottom to −104 mm along the *X*‐direction, representing a maximally offset configuration. Conversely, step #18 presents the mirrored arrangement. These examples demonstrate the planned repositioning strategy used to improve angular coverage and image quality throughout the field of view.

### 2.3 System matrix and LOR definitions

To model each line of response (LOR) in the system matrix, we adopted a multi‐ray sampling strategy that accounts for photon interaction variability within the detector crystals. Instead of assuming a single ideal path between the centers of opposing crystals, each LOR i was represented by a set of C rays defined by sampled pairs of interaction points. These points, denoted as $v_{0}^{\left(k\right)}=\left(x_{0}^{\left(k\right)},y_{0}^{\left(k\right)},z_{0}^{\left(k\right)}\right)$ in the first crystal and $v_{1}^{\left(k\right)}=\left(x_{1}^{\left(k\right)},y_{1}^{\left(k\right)},z_{1}^{\left(k\right)}\right)$ in the second crystal, define the path of ray *k*, where k=1,2,…,C.

The length of each ray $L_{k}$ is computed as the Euclidean distance between $v_{0}^{\left(k\right)}$ and $v_{1}^{\left(k\right)}$. For each ray k, the attenuation factor $a_{d}^{\left(k\right)}$ represents the combined attenuation within the two detector crystals defining each LOR, accounting for the photon path lengths inside both crystals; $while\;l_{j}^{\left(k\right)}$ denotes the intersection length of the ray within voxel j.

The geometric system matrix element $g_{ij}$, representing the contribution of voxel j to LOR i, is given by:

(1) $$g_{ij}\approx \frac{1}{C}\sum_{k=1}^{C}\frac{a_{d}^{\left(k\right)}}{L_{k}^{2}}\cdot l_{j}^{\left(k\right)}$$

This formulation averages the contributions from all rays assigned to each LOR, where each ray is weighted by detector attenuation and an inverse‐square geometric factor, which accounts for the angular spread and distance‐dependent sensitivity of each ray path. This approach enhances the accuracy of the system matrix, particularly in capturing depth‐of‐interaction effects and finite detector resolution in a layered detector.^30^, ^31^

### 2.4 List‐mode image reconstruction

Two reconstruction approaches were implemented and compared: the baseline list‐mode time‐of‐flight maximum likelihood expectation maximization (MLEM) algorithm, and a stochastic primal–dual hybrid gradient algorithm with total variation (TV) regularization, referred to as the hybrid method in this study.^32^ Both methods used the same geometric system matrix described in Section 2.2, and reconstruction was performed on identical list‐mode datasets with TOF information. The MLEM algorithm serves as the standard statistical baseline, while the hybrid method incorporates non‐smooth regularization within a memory‐efficient, subset‐based optimization framework.

#### 2.4.1 Classical MLEM algorithm

Baseline image reconstruction was performed using a classic list‐mode TOF‐MLEM algorithm extended to a multi‐ray formulation. Each LOR was represented by a set of sampled rays, and each ray was modelled individually using inverse‐square geometric weighting and TOF kernel integration. The voxel update at iteration *n+1* follows the classical MLEM formulation and is given by:

(2) $$\begin{array}{cc} f_{j}^{\left(n+1\right)} & =f_{j}^{\left(n\right)}\cdot \left(\frac{1}{\sum_{i}\sum_{r=1}^{C}g_{ij}^{\left(r\right)}}\right)\\ & \;\cdot \sum_{i}\sum_{r=1}^{C}\left[g_{ij}^{\left(r\right)}\cdot \left(\frac{1}{C\cdot \sum_{k}g_{ik}^{\left(r\right)}f_{k}^{\left(n\right)}}\right)\right] \end{array}$$

where $f_{j}^{\left(n\right)}$ is the estimated activity in voxel j at iteration n, $g_{ij}^{\left(r\right)}$ is the system matrix element relating voxel j to the r ^th^ ray of LOR i, and each LOR is modeled by C rays. The contribution of the measured event is evenly distributed across its rays using a weighting factor of $1/(C\cdot \sum_{k}g_{ik}^{\left(r\right)}f_{k}^{\left(n\right)})$. A total of 60 iterations were performed for MLEM reconstructions. The time‐of‐flight resolution was set at 180ps. The voxel grid was defined as 0.75×0.75×0.75 $mm^{3}$ over a field‐of‐view (FOV) of $120\times 150\times 105\;{\mathrm{mm}}^{3}$, resulting in a reconstructed image size of 160×200×140 voxels. The evaluation of different ray‐sampling configurations was performed using the scaled NEMA image‐quality phantom simulation described in Section 2.7.2.

To evaluate the effect of geometric modeling, the reconstruction was performed using six different ray sampling strategies: one ray per LOR, and multi‐ray models with 4, 8, 16, 32, and 64 rays per LOR. For each ray, the photon interaction point within the detector crystal were stochastically selected within the detector crystal volume. A uniform distribution was used along the depth of the crystal, while a Gaussian blur of 3.5 mm FWHM was applied to reflect the experimentally measured axial resolution when sampling interaction points along the *Z*‐direction. For the single‐ray configuration, one interaction point was randomly selected within each detector crystal, consistent with the stochastic sampling scheme used in the multi‐ray model but limited to a single path per LOR. In the list‐mode implementation, TOF information is incorporated event‐wise during forward and back‐projection rather than pre‐computed in the system matrix. For each detected event, a Gaussian TOF kernel centered at its measured timing offset is evaluated on‐the‐fly along the corresponding LOR to weight voxel contributions. The normalization factors, obtained from a uniform flat phantom, are TOF‐independent and correct for geometric and detector‐efficiency variations. The normalization matrix used in the MLEM algorithm was obtained from a Monte Carlo simulation of a uniform flat phantom with a thickness of 1mm, which incorporates detector efficiency and geometric effects.^27^

After preliminary evaluation, the configuration using 16 rays per LOR and 35 MLEM iterations was selected and fixed for all subsequent reconstructions, providing a balance between reconstruction fidelity and computational efficiency.

#### 2.4.2 List‐mode hybrid reconstruction

We employed the list‐mode hybrid algorithm, a stochastic primal–dual hybrid gradient (SPDHG), to reconstruct PET images directly from list‐mode data while incorporating non‐smooth regularization. The method minimizes a regularized negative Poisson log‐likelihood cost function, which balances data fidelity with prior information using the result output of the standard MLEM utilizing 16‐rays. The objective function is defined as:

(3) $$\min_{x\geq 0}\sum_{e}\left({\left(Ax+s\right)}_{e}-\log\left({\left(Ax+s\right)}_{e}\right)\right)+\beta \cdot R\left(Kx\right)$$

In this equation, x is the image to be estimated, the system matrix A models the forward projection from image space to list‐mode event space, with its elements defined as $A_{ij}=g_{ij}$ in Equation 2, and s is a correction term for contamination (randoms and scatter). The regularization term R(Kx) promotes desirable image properties (such as sparsity or smoothness), where K is a linear operator (typically the image gradient), and β controls the strength of the prior. The algorithm updates the image x and dual variables through a stochastic primal–dual scheme. The list‐mode data is divided into n subsets, and at each iteration, one subset is selected at random for update. This enables efficient processing with reduced memory footprint.^32^ In this study, we used 28 subsets with 100 iterations. The regularization parameter β=0.1 was selected empirically to balance noise suppression and contrast recovery. Preconditioned step sizes are computed separately for each data subset s, using:

(4) $$S_{s}=\gamma \cdot \mathrm{diag}\left(\frac{\rho }{A_{s}}\right)$$

(5) $$T_{s}=\gamma^{-1}\cdot \mathrm{diag}\left(\frac{\rho \cdot p_{s}}{A_{s}^{\mathrm{tr}}/\mu_{s}}\right)$$

(6) $$T=\min_{s=1,\text{…},n}T_{s}$$

Here, γ is a positive scaling parameter set to 1.1. The matrix $A_{s}$ denotes the submatrix of the system matrix corresponding to the subset of LORs included in subset s. The constant ρ is a normalization factor between 0 and 1 (set to 0.9 in this study), and $p_{s}$ is the probability of selecting subset s. The operator tr denotes transpose, and $\mu_{s}$ is the expected event count in subset s. The final step size T is defined as the element‐wise minimum across all subsets. Total variation (TV) was used as the prior, implemented using first‐order forward differences and the mixed L1–L2 norm.^33^ The proximal operator of the convex conjugate of the TV functional was applied in the dual update. This choice enables edge‐preserving regularization without over‐smoothing fine details. The algorithm was initialized with a single iteration of MLEM to warm start both the image and the dual variables.

This reconstruction strategy enables memory‐efficient, GPU‐compatible processing of high‐resolution PET images from list‐mode data, while supporting modern priors for resolution recovery and noise suppression.

### 2.5 Resolution modeling

Due to the limited angular coverage of the dual‐panel PET geometry, the reconstructed images exhibit strong anisotropic point spread function (PSF) behavior.^34^ The elongation of point sources is spatially dependent, with minimal distortion near the center of the FOV and increasingly severe elongation near the edges and corners. These spatially varying resolution effects are caused by the limited‐angle geometry, the presence of module gaps, and overlapping acquisition steps, leading to complex shift‐variant degradation patterns across the image volume.

**FIGURE 3:**
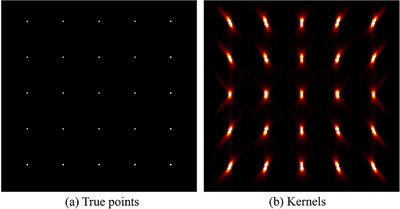
Transverse view of (a) the simulated grid of point sources used for PSF kernel extraction and (b) the corresponding reconstructed PSFs after two MLEM iterations, showing spatially variant elongation patterns due to the dual‐panel geometry and limited‐angle acquisition.MLEM, maximum likelihood expectation maximization.

To address this, a shift‐variant PSF modeling approach was developed.^35^ A regular grid of PSF kernels was generated using Monte Carlo simulations. Specifically, 5×5×5 point sources (with 1mm diameter) were simulated at uniformly spaced positions separated by 21mm in each direction, covering the full FOV. Figure 3 illustrates the true source locations, and the corresponding deformed reconstructed images obtained after two iterations of the MLEM algorithm described in Section 2.4.1.

Each reconstructed $21\times 21\times 21\;{\mathrm{mm}}^{3}$ sub‐volume centered on the simulated point location was extracted and used as a PSF kernel. These kernels capture the spatially varying elongation and blurring characteristics, which are later applied in a convolutional processing step to enhance resolution. Prior to PSF extraction, a median filter was applied to the reconstructed image before PSF extraction to suppress high‐frequency noise and avoid kernel artifacts (as shown in Figure 3b).

Shift‐variant PSF kernels were extracted directly from Monte Carlo–simulated point‐source reconstructions across the full FOV, without background activity, to isolate the intrinsic system response. Each kernel represents the locally measured system PSF, capturing anisotropic elongation and blurring patterns. This modeling introduced a trade‐off between elongation reduction and minor Gibbs‐like edge artifacts; therefore, in the Results section, the configuration providing the optimal balance between sharpness and minimal artifact was reported for MLEM‐PSF reconstruction.

During PSF modeling, each voxel update is performed using a locally adapted kernel constructed through a weighted interpolation of the four nearest precomputed PSF kernels. Let *x* denote the voxel position and let $x_{1},x_{2},x_{3},x_{4}$ be the positions of the four nearest kernel centers. The interpolated kernel $K_{x}$ is defined as $K_{x}=w_{1}\;\left(x\right)K_{1}+w_{2}\left(x\right)K_{2}+w_{3}\left(x\right)K_{3}+w_{4}\left(x\right)K_{4}$ where $K_{i}$ is the PSF kernel centered at $x_{i}$, and $w_{i}\left(x\right)$ is the spatial interpolation weight for voxel x with respect to the kernel at $x_{i}$, such that $w_{1}\left(x\right)+w_{2}\left(x\right)+w_{3}\left(x\right)+w_{4}\;\left(x\right)=1$. The interpolation weights $w_{i}\left(x\right)$ were calculated using inverse‐distance weighting among the four nearest kernel centers, such that closer kernels contribute more strongly. This approach ensures smooth transitions and avoids visible boundaries between adjacent kernel regions. This interpolation ensures smooth kernel variation across space. Each voxel is then updated through a convolutional operation using the interpolated kernel $K_{x}$, resulting in a locally elongated version of the image:

(7) $$f^{\prime }=f*K_{x}$$

where f is the original image, $K_{x}$ is the voxel‐specific kernel, and ∗ denotes 3D convolution. The resulting image $f^{\prime }$ preserves the spatially variant blurring pattern and is subsequently used as the source image for the forward projection step in each iteration of the reconstruction algorithms described in Section 2.4.1. The interpolated PSF kernel was applied to the image estimate before the forward projection step to model spatially variant elongation patterns. This operation enables the algorithm to iteratively compensate for system blurring, progressively reducing the effective elongation across iterations.

### 2.6 Deep learning‐based resolution recovery

To further improve resolution and contrast recovery, we implemented a deep learning‐based post‐processing pipeline using a 3D Swin‐UNET architecture^36^ trained to enhance MLEM‐reconstructed images. The network was designed to correct elongation artefacts not fully resolved by model‐based reconstruction alone.

#### 2.6.1 Dataset generation

A total of 294 3D image pairs were generated for training and validation. Ninety‐seven volumes were extracted from clinical ^68^Ga‐PSMA (Gallium‐68–labeled Prostate‐Specific Membrane Antigen, PSMA) PET acquisitions on full‐ring PET/CT systems (49 volumes on Siemens Biograph mCT and 48 volumes on Siemens Biograph Vision), anonymized and pre‐processed to focus on the pelvic region, serving as clean reference images free of elongation artefacts. Each volume was randomly rotated along one or two axes (up to ± 40 degrees), and the FOV was randomly shifted to augment anatomical diversity. This process doubled the real dataset to 194 synthetic‐real samples. An additional 100 synthetic 3D volumes were generated, each containing randomly placed, abstract geometric shapes (e.g., spheres, rectangles, triangles) with varying sizes, depths, and orientations in 3D space. These were used to enforce generalizability and spatial diversity in the model. For all volumes, list‐mode data were generated using the dual‐panel scanner model and reconstructed using standard MLEM. This reconstruction step naturally introduced the elongation and geometry‐dependent artefacts characteristic of the system, which served as the degraded inputs for training, while the original activity maps were used as supervision labels.

#### 2.6.2 Model architecture and training

The network architecture was based on the Swin UNET model, implemented in Torch. It accepts a single‐channel 3D image as input and outputs a single‐channel enhanced image. The core transformer‐based encoder–decoder architecture was configured with a patch size adapted to the voxel size of the PET data and feature size of 48. The model was trained using the Adam optimizer. A one‐cycle learning rate scheduler with cosine annealing and momentum cycling was applied to encourage stable convergence. Training was conducted over 100 epochs and a validation split of 10%. A custom shape‐sensitive loss function was employed, combining weighted MSE, MAE, and a structural component to emphasize lesion edges. The loss weights (α, β, γ) were dynamically adjusted during training using a cosine‐annealed scheduling strategy defined by the training step. These weights began with equal emphasis and gradually shifted to prioritize fine‐detail recovery (γ) over global error (α), while keeping the smoothness term (β) small but stable. Training utilized mixed‐precision and gradient clipping for stability. Training performance was monitored using MSE, MAE, and PSNR validation metrics. The explicit formulation of the composite loss function combining weighted MSE, MAE, and structural similarity terms is provided in the Supporting Information (S1).

### 2.7 Monte Carlo simulations

Monte Carlo simulations were conducted using the Geant4 Application for Tomographic Emission (GATE) platform,^37^ with full modeling of the Provision scanner's dual‐panel geometry, including four‐layer depth‐of‐interaction detector modules, inter‐module gaps, time‐of‐flight resolution (180ps FWHM), and energy resolution (14% FWHM at 511 keV). The exact physical scanner configuration, including the 3 × 4 detector module layout per panel and 8.8 mm/7.1 mm inter‐module spacing (*X*/*Z*), was reproduced in detail.

A fixed inter‐panel distance of 274 mm was used for all simulations. Each simulation employed the same acquisition protocol used throughout this study, consisting of 22 predefined detector panel positions (as described in Section 2.2). These positions were selected to optimize angular coverage by systematically shifting the top and bottom panels in the *X* and *Z* directions. Simulation outputs were stored in list‐mode format for consistency with reconstruction inputs.

#### 2.7.1 Grid‐based point source simulation

A 5 × 5 × 5 grid of point sources was simulated to generate spatially variant PSF kernels for use in resolution modeling and post‐reconstruction deconvolution. Each point source was modeled as a 1.0 mm‐diameter sphere filled with low‐intensity positron‐emitting activity and spaced uniformly at 21 mm intervals in all three dimensions. The goal of this simulation was to characterize the spatial dependence of image blurring across the FOV due to limited‐angle geometry. For each point, the full dual‐panel acquisition was simulated using the 22‐step protocol, and reconstructed images were used to extract local PSF kernels as described in Section 2.5.

#### 2.7.2 Scaled NEMA image quality phantom simulation

A second Monte Carlo simulation was performed using a scaled‐down version of the NEMA image quality phantom, adapted to the limited FOV of the Provision system.^38^, ^39^ The phantom consisted of a cylindrical background volume (98.4 mm diameter) containing six spherical inserts with diameters of 3, 4, 5.5, 7.5, 10, and 13 mm. The spheres were filled at a 10:1 activity ratio relative to the background, with a total injected activity of 2.5 MBq.

This simulation also used the full 22‐step acquisition protocol under the same scanner geometry and physical setup. The resulting dataset served as the primary input for evaluating reconstruction performance under different algorithmic configurations, as described in Section 2.7.

### 2.8 Experimental phantoms

To evaluate reconstruction performance under realistic anatomical conditions, we conducted an experimental scan using the Adam–Peter anthropomorphic phantom.^40^ The phantom replicates the male pelvic anatomy, including compartments for soft tissue, prostate, bladder, and lesions. All compartments were filled with ^18^F with different concentrations. The main body of the phantom was filled with oil to simulate soft‐tissue attenuation. The bladder compartment was filled with an activity concentration of 10 kBq/mL, while the prostate region was filled with 170 kBq/mL. Two spherical lesions were embedded in the posterior section of the prostate: Lesion #1 (8 mm diameter) was filled with 1000 kBq/mL, whereas Lesion #2 (10 mm diameter) was filled with 2000 kBq/mL. The data were reconstructed using four different algorithms: the standard list‐mode MLEM, MLEM with embedded shift‐variant PSF modeling (MLEM‐PSF), the hybrid list‐mode, and the Swin‐based deep‐learning enhancement.

**FIGURE 4:**
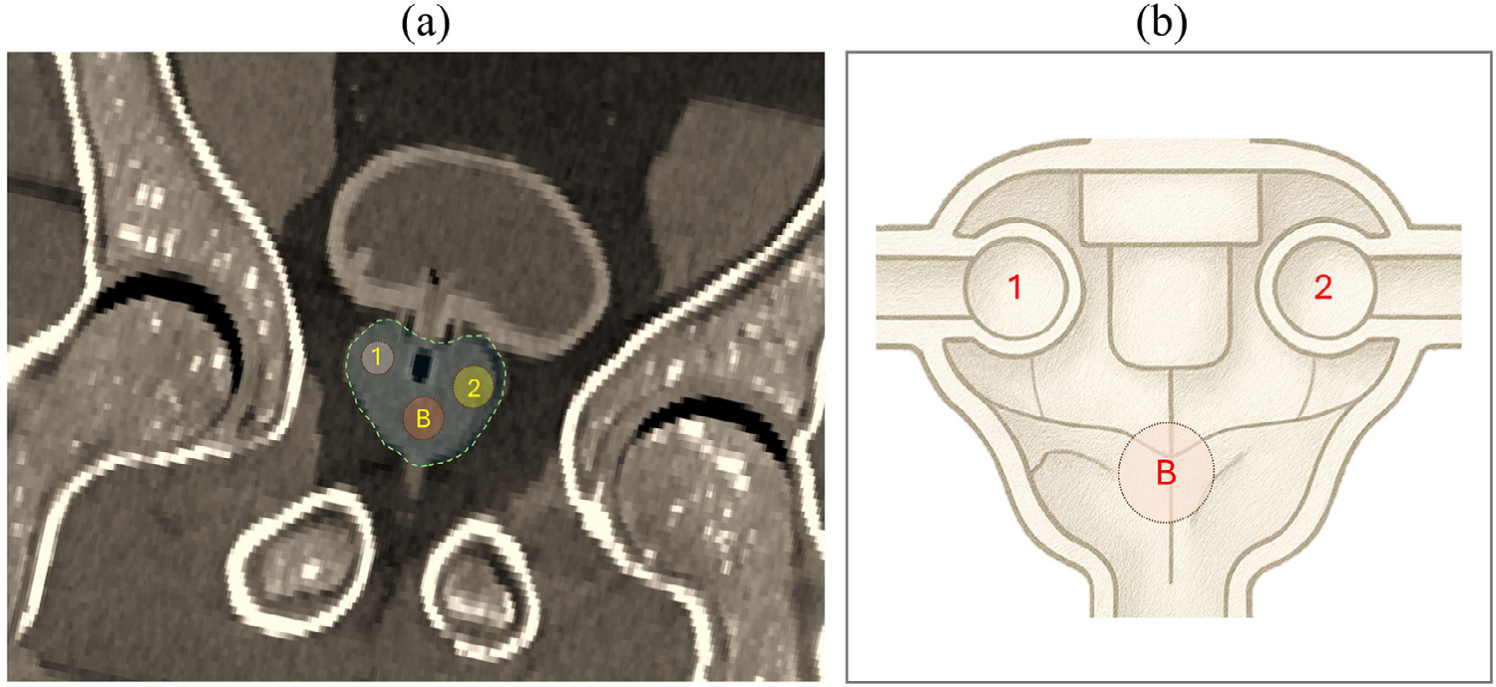
(a) CT slice of the Adam–Peter anthropomorphic phantom showing the prostate region with annotated volumes of interest (VOIs): two spherical lesions and background. (b) Schematic representation of the prostate lobes illustrating the spatial layout of the three VOIs. Lesion #1 and Lesion #2 were defined with diameters of 8 and 10 mm, respectively, whereas the background VOI was defined using a 10 mm spherical volume within the prostate compartment.

This configuration corresponded to lesion‐to‐prostate contrast ratios (target CNC values) of approximately 5.88 for Lesion #1 and 11.76 for Lesion #2. Spherical volumes of interest (VOIs) were defined with diameters matching the actual structures: 8 mm for Lesion #1, 10 mm for Lesion #2, and 10 mm for the background region placed in the prostate. The 22‐position acquisition protocol described in Section 2.2 was used, and the data were reconstructed using three different algorithms. Evaluation focused on quantifying lesion detectability and signal recovery relative to the known activity ratios (Figure 4).

### 2.9 Evaluation metrics

To assess reconstruction performance across simulated and experimental datasets, standard image quality metrics were employed. Background noise was measured using the coefficient of variation (CoV) calculated by dividing the standard deviation of voxel intensities by the mean value within a uniform background volume of interest (VOI, Equation 8). This value, expressed as percentage, reflects the relative noise level in background areas.

(8) $$\mathrm{CoV}=\frac{\sigma_{B}}{\mu_{B}}$$

CNR was used to evaluate the detectability of high‐uptake regions. For each hot sphere or lesion, the CNR was calculated by taking the difference between the mean activity in the lesion and the background, divided by the standard deviation of the background region (Equation 9). A higher CNR indicates better visibility and distinction of the lesion from background noise.

Contrast recovery coefficient (CRC) was also computed to quantify uptake recovery. CRC is defined as the ratio of the mean activity in the lesion VOI to that in the background VOI (Equation 10). In this study, the largest lesion was used for CRC measurements to ensure stability and minimize partial volume effects.

To further assess image consistency, we calculate CNC, the contrast‐to‐noise consistency which is defined as normalized by the standard deviation of the background (Equation 11). CNC provides a normalized contrast metric that reflects intensity preservation while accounting for overall image brightness. It complements CNR by focusing on signal‐level consistency rather than noise separation alone.

The following formulas were used to compute the image quality metrics:

(9) $$\mathrm{CNR}=\frac{|\mu_{L}-\mu_{B}|}{\sigma_{B}},$$

(10) $$\mathrm{CRC}=\frac{\mu_{L}/\mu_{B}}{C_{true}},$$

(11) $$\mathrm{CNC}=\frac{\mu_{L}}{\mu_{B}},$$

where $\mu_{L}$ and $\mu_{B}$ denote the mean activity within the lesion and background VOIs, $\sigma_{B}$ is the standard deviation of the background, and $C_{true}$ is the known true concentration ratio.

All evaluations were conducted using the 22‐step acquisition protocol described in Section 2.2, under a fixed inter‐panel distance of 274 mm. Four datasets were used for this study: the PSF grid simulation with 125‐point sources arranged in a 5 × 5 × 5 pattern, the scaled NEMA phantom simulation with six spheres of varying diameters, and the Adam–Peter anthropomorphic phantom filled with ^18^F solution.

For all reconstructions, the spherical VOIs were defined using the true physical dimensions of the phantom spheres rather than their visually elongated shapes in the reconstructed images, ensuring consistent evaluation across methods in line with NEMA standards.

## 3 RESULTS

### 3.1 Multi‐ray sampling optimization

To investigate the influence of ray sampling on reconstruction quality, we systematically evaluated the multi‐ray MLEM algorithm using six different ray count strategies per LOR: 1, 4, 8, 16, 32, and 64. Each configuration was reconstructed using up to 60 MLEM iterations.

**FIGURE 5:**
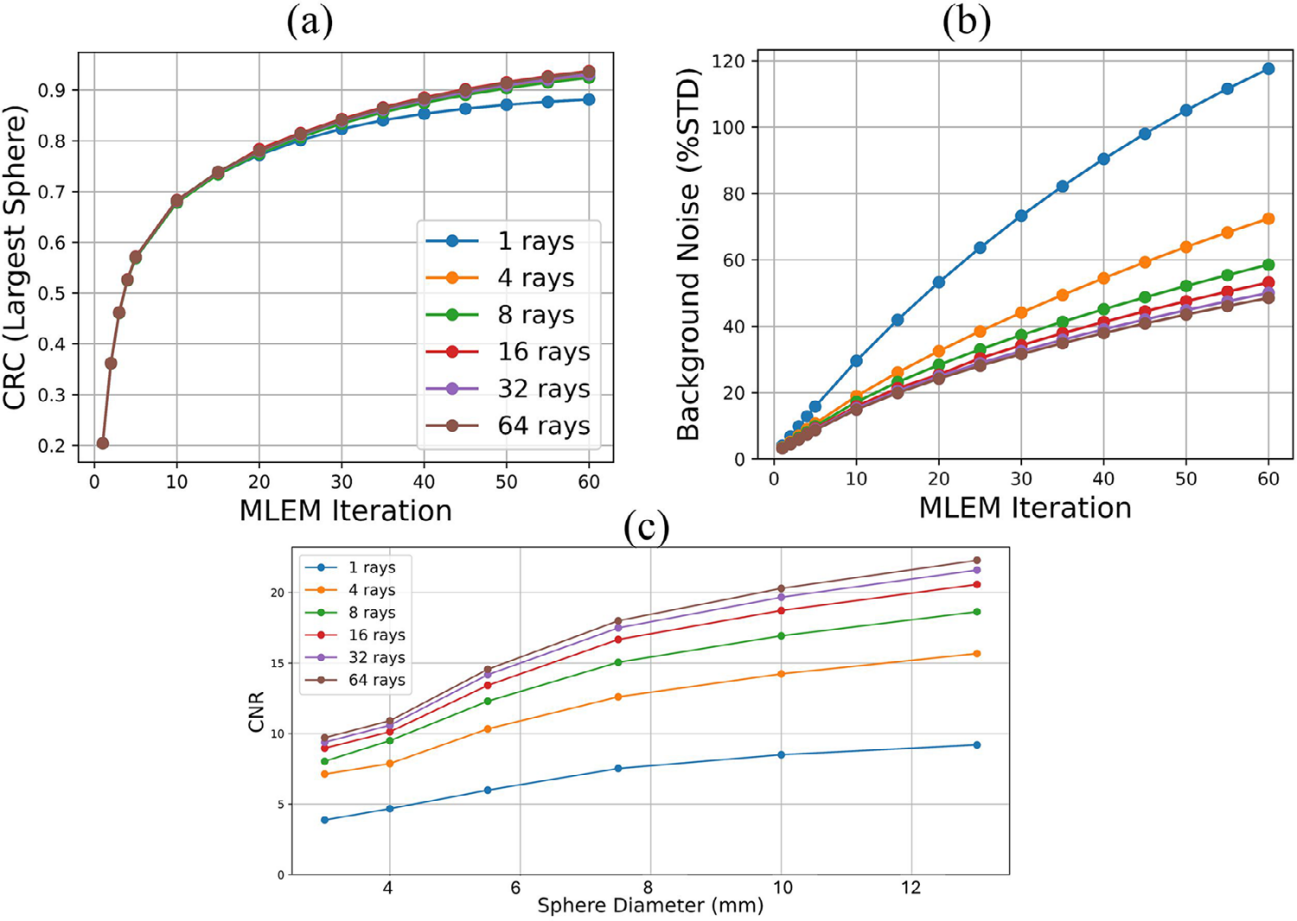
Quantitative comparison of reconstruction performance for different ray counts per LOR using the scaled‐down NEMA phantom. All configurations (1, 4, 8, 16, 32, and 64 rays) were computed, the 4‐ray curve overlaps with the 8‐ and 16‐ray curves because of their similar performance. (a) Contrast recovery coefficient (CRC) for the largest sphere plotted across MLEM iterations. Higher ray counts lead to improved CRC and faster convergence. (b) Background noise, expressed as coefficient of variation (%STD), increases with iteration and is significantly higher for the 1‐ray configuration. (c) Contrast‐to‐noise ratio (CNR) as a function of sphere diameter. Multi‐ray configurations show clear CNR gains, particularly for smaller spheres. Gains saturate beyond 16 rays. LOR, line of response.

Figure 5 summarizes the quantitative evaluation results. In Figure 5a, the CRC for the largest sphere is plotted as a function of iteration number. All multi‐ray configurations show increasing CRC with iteration, but configurations with more rays consistently reached higher CRC values. The curve for 1‐ray reconstruction plateaus at a lower value, indicating degraded recovery due to insufficient angular integration.

Figure 5b shows the background noise, measured as the coefficient of variation in a central background region. As expected, noise increases with iteration. The 1‐ray configuration exhibits significantly higher noise levels compared to the multi‐ray cases. After approximately 35 iterations, minor separation between the 16‐ray and higher‐ray configurations can be observed, although their overall performance trends remain similar.

Figure 5c illustrates the CNR across different sphere diameters at a fixed iteration. CNR improves consistently with ray count, with a particularly large gap between 1 ray and multi‐ray configurations. Performance increases moderately between 4 and 16 rays but saturates between 16 and 64 rays, indicating that 16 rays offer a reasonable balance between image quality and computational cost.

**FIGURE 6:**
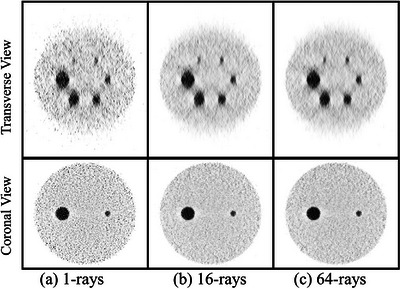
Transverse (top row) and coronal (bottom row) slices of the scaled‐down NEMA phantom reconstructed using (a) 1 ray, (b) 16 rays, and (c) 64 rays per LOR. All images are shown at 35 iterations. The 1‐ray reconstruction shows elevated noise and degraded small‐sphere visibility, while the 16‐ray and 64‐ray reconstructions achieve improved contrast and uniformity, with minimal visual differences between them. LOR, line of response.

Figure 6 presents transverse and coronal slices of the scaled‐down NEMA phantom reconstructed using 1, 16, and 64 rays per LOR, all at iteration 35. The 1‐ray reconstruction shows substantial noise and blurred sphere boundaries. In contrast, the 16‐ray and 64‐ray images both exhibit sharper sphere boundaries of the hot spheres and smoother background. Visual differences between 16 and 64 rays are minimal, confirming the diminishing returns of using a higher number of rays.

### 3.2 Comparison of reconstruction algorithms

**FIGURE 7:**
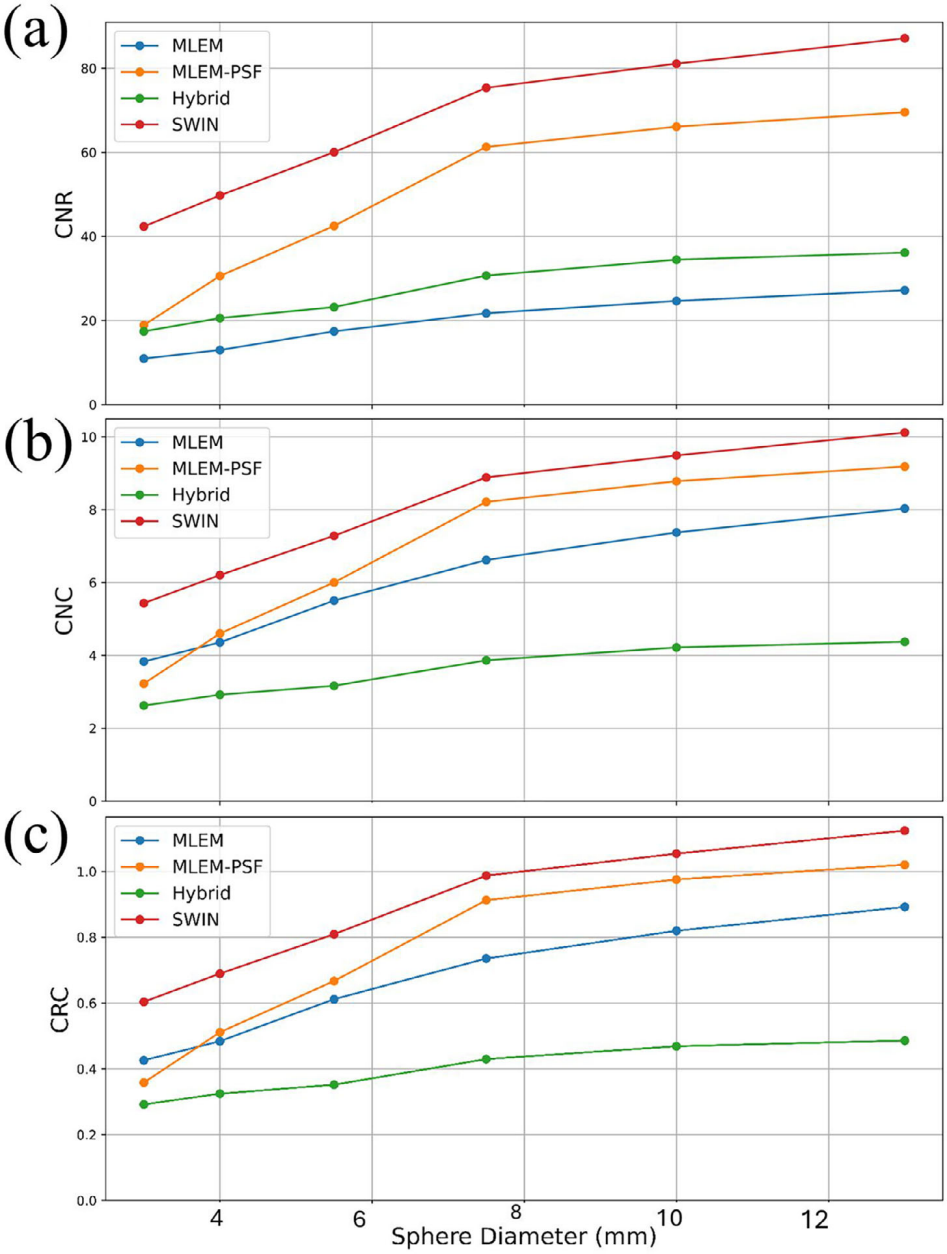
Quantitative evaluation of reconstruction methods on the scaled NEMA phantom. (a) Contrast‐to‐noise ratio (CNR) plotted versus sphere diameter. (b) Contrast‐to‐noise consistency (CNC) computed as CNR normalized by background noise. (c) Contrast recovery coefficient (CRC) showing quantitative recovery across sphere sizes. Error bars represent ± 1 SD across three repeated reconstructions.

To evaluate the influence of reconstruction strategy on image quality, we compared four approaches using the scaled NEMA phantom: the classic list‐mode MLEM algorithm; MLEM with embedded shift‐variant PSF modeling (MLEM‐PSF); the hybrid list‐mode reconstruction; and a Swin‐based 3D deep learning model (SWIN) applied as a post‐processing step to MLEM‐reconstructed images. All reconstructions were performed using the same 16‐ray geometric model and 22‐step acquisition protocol unless otherwise noted. Quantitative results are summarized in Figure 7, which presents CNR, CNC, and CRC across sphere sizes for all methods. The SWIN model achieved the highest and most consistent performance across these metrics, maintaining high CRC values comparable to the ground‐truth activity ratios. MLEM‐PSF also improved CNR and CRC relative to standard MLEM owing to its resolution modeling, while the hybrid method yielded intermediate performance between the physics‐based and learning‐based approaches.

**FIGURE 8:**
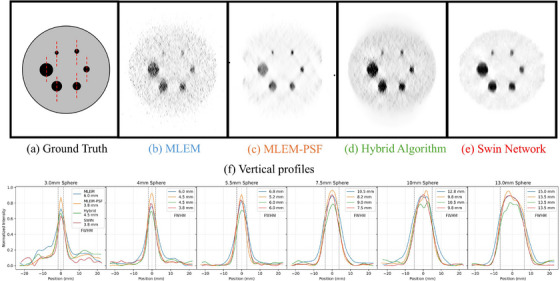
Ground‐truth phantom and reconstructed images using different algorithms, with corresponding vertical profiles. (a) Ground‐truth phantom geometry with profile positions (dashed red lines). (b) MLEM, (c) MLEM‐PSF, (d) hybrid algorithm, and (e) SWIN network reconstructions. (f) Normalized vertical intensity profiles through each sphere (3–13 mm diameter). FWHM values shown in the plots indicate that MLEM‐PSF and SWIN most closely reproduce the true sphere dimensions, hybrid yields intermediate sharpness, while MLEM shows the largest axial elongation. MLEM, maximum likelihood expectation maximization.

Figure 7b presents the CNC, defined as CNR normalized by the background standard deviation. The SWIN network again outperformed the other methods, indicating its ability to enhance contrast without amplifying background noise. While MLEM‐PSF achieved strong CNR, its increased background variability reduced overall CNC. The hybrid method maintained low background noise but at the cost of reduced lesion contrast. Figure 8 presents the reconstructed phantom images and quantitative vertical‐profile analysis. The SWIN reconstruction (Figure 8e) provides the most uniform background and highest lesion sharpness, yielding FWHM values closest to the ground truth. MLEM‐PSF (Figure 8c) improves structural recovery relative to MLEM (Figure 8b) but introduces mild texture patterns. The hybrid algorithm (Figure 8d) achieves smoother noise characteristics but slightly softer lesion edges. As shown on the profiles in Figure 8f, SWIN and MLEM‐PSF achieve the narrowest FWHM across all spheres, confirming reduced axial elongation compared with the conventional MLEM reconstruction.

These results demonstrate the benefits of combining accurate system modeling with learned post‐processing. While PSF modeling improves resolution through physical description of the imaging system, the deep learning approach achieves superior lesion detectability and consistency by learning spatially variant corrections from data.

### 3.3 Experimental phantom studies

**FIGURE 9:**
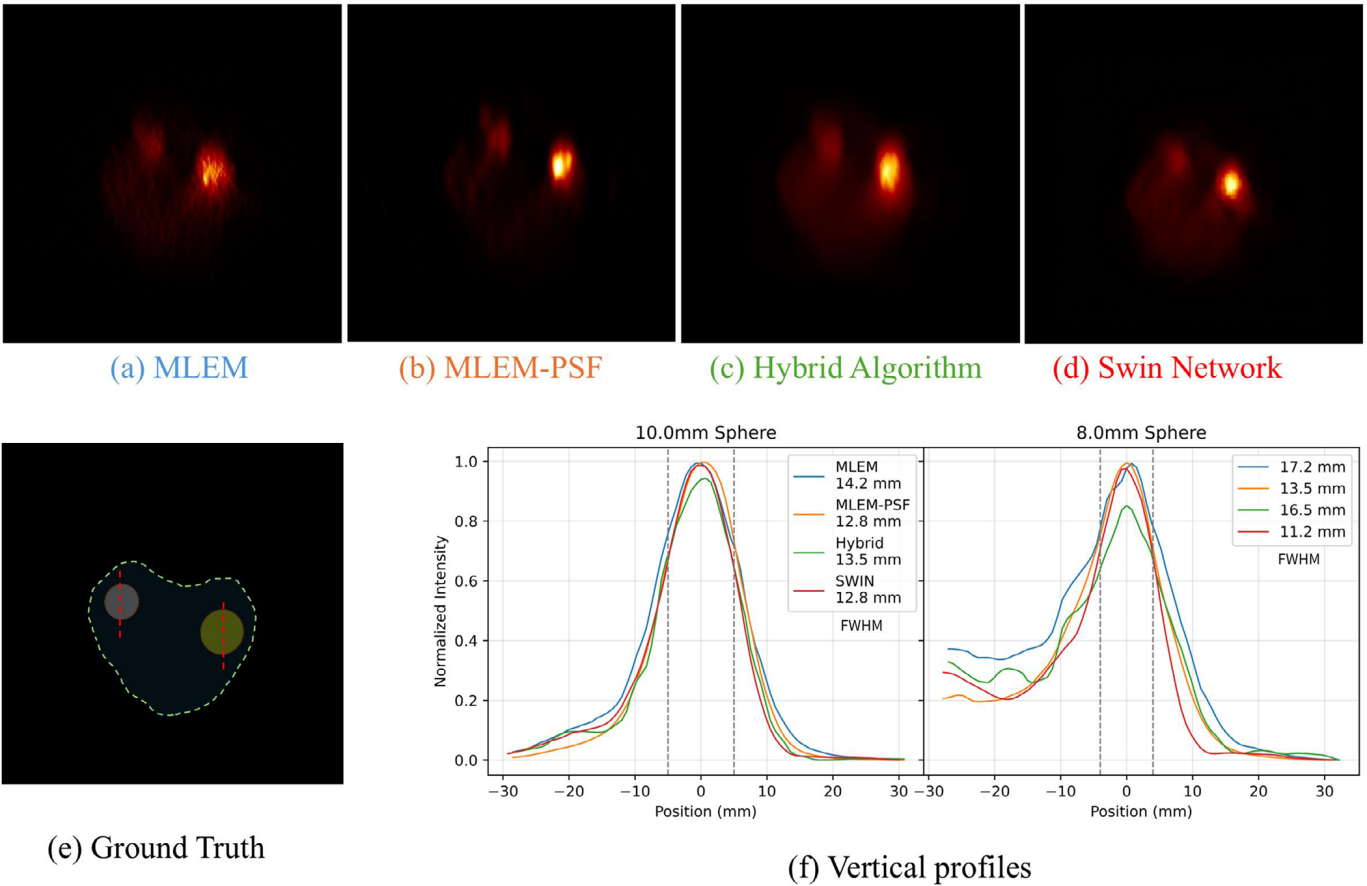
Transverse slices of the Adam–Peter phantom reconstructed using (a) MLEM, (b) MLEM‐PSF, (c) hybrid algorithm, and (d) Swin network. (e) Ground‐truth phantom geometry with vertical‐profile locations (dashed red lines). (f) Normalized vertical line profiles through the 10 and 8 mm spheres showing reduced elongation for the MLEM‐PSF and Swin methods (FWHM values indicated). MLEM, maximum likelihood expectation maximization.

Experimental validation was performed using the Adam–Peter anthropomorphic phantom described in Section 2.8. Figure 9 presents the transverse PET slices, corresponding ground‐truth geometry, and vertical line profiles through the lesion centers.

The MLEM reconstruction (Figure 9a) displays visible background noise and moderate blurring. The MLEM‐PSF result (Figure 9b) improves spatial recovery and reduces elongation artefacts. The hybrid algorithm (Figure 9c) yields smoother background with slightly softer edges, while the Swin network (Figure 9d) achieves the cleanest background and highest lesion contrast. The profile plots (Figure 9f) demonstrate that MLEM‐PSF and Swin most closely reproduce the true sphere dimensions, yielding FWHM values of 12.8 mm for the 10 mm sphere and 11.2 mm for the 8 mm sphere.

Quantitatively, the CNR for the larger lesion was 10.43 (MLEM), 14.48 (Hybrid), and 13.85 (Swin); for the smaller lesion 2.28, 3.03, and 4.35, respectively. These results confirm that while the hybrid approach enhances noise uniformity, the MLEM‐PSF and Swin methods provide superior spatial resolution and lesion delineation.

## 4 DISCUSSION

In the Provision PET scanner, the dual‐panel PET detectors provide limited angular coverage, which poses challenges for artifact‐free image reconstruction. Despite employing PSF modeling within the MLEM algorithm, the elongation artifact persisted. PSF kernels were accurately estimated in a shift‐variant manner using a grid source and Monte Carlo simulations, accounting for the system's spatial response. However, the highly anisotropic nature of the PSF kernels, particularly as one moves away from the center of the FOV, resulted in visible image artifacts during PSF‐based reconstruction. Although the shift‐variant PSF kernels derived from Monte‐Carlo simulations were incorporated into the reconstruction to model the spatially varying system response, residual elongation persisted because the limited angular coverage of the dual‐panel geometry cannot be fully compensated by PSF modeling alone. In this work, PSF modeling was implemented in image space to provide a reference for evaluating elongation‐reduction strategies, consistent with prior limited‐angle PET studies. While resolution modeling could be directly embedded in the system matrix, image‐space formulation was implemented to compare representative reconstruction strategies rather than optimizing a full resolution‐modelled framework. To mitigate the elongation artifacts caused by limited angular coverage in the Provision PET scanner, we developed a post‐reconstruction deep‐learning‐based correction approach using a Swin UNETR architecture. The network was trained on realistic MLEM reconstructions of ^68^Ga‐PSMA patient data, augmented with synthetic patterns to reflect scanner‐specific degradation. While the elongation was not fully corrected, the method provided the most substantial improvement among all tested approaches. In both scaled NEMA and anthropomorphic phantom experiments, it achieved the highest CNR and CNC. These findings highlight the effectiveness of data‐driven enhancement in addressing geometric artifacts where physical modeling alone is insufficient.

For the experimental results, since the elongation artefact in this system is dominated by the dual‐panel geometry, and ^68^Ga has a larger positron range than ^18^F, the ^68^Ga–based training data represent a slightly more blurred case than the ^18^F phantom images, making this a conservative training‐to‐inference scenario. Moreover, because the PET volumes in this study covered a limited pelvic region containing only the prostate, the uptake patterns between FDG and PSMA radiotracers did not differ substantially.

The multi‐ray MLEM algorithm increases computational cost approximately in proportion to the number of sampled rays, but image‐quality improvements saturate beyond 16 rays per LOR (Figure 5). This configuration therefore provides a practical balance between image quality and runtime and remains compatible with GPU‐based implementations in current research prototypes. The hybrid reconstruction achieved lower background noise and stable contrast but produced slightly smoother lesion boundaries compared with MLEM and SWIN. It is best suited for low‐count acquisitions or situations where noise suppression is prioritized. Further development could explore combinations of physical modeling, regularized optimization, and data‐driven enhancement to unify the complementary strengths of these reconstruction strategies.

This approach aligns with prior work by Raj et al.,^11^ who proposed a similar deep‐learning‐based post‐reconstruction elongation correction for a dual‐panel PET scanner, utilizing a training dataset created by applying shift‐variant PSF kernels obtained from grid reconstructions of point sources to random patterns. In contrast, our training dataset was generated using a novel list‐mode reconstruction of real patient images, complemented by random pattern modeling that precisely replicated the scanner's geometry, angular coverage, noise distribution, resolution characteristics, and reconstruction artifacts. By directly incorporating elongation, noise distribution, and other image artifacts observed in actual PET scanner reconstructions, our list‐mode‐based training dataset offers a more comprehensive representation of the imaging conditions compared to the kernel‐based approach employed by Raj et al.^11^ We believe this method enhances the accuracy of elongation artifact modeling, leading to improved correction performance in the deep‐learning framework.

## 5 CONCLUSION

This study evaluated image reconstruction strategies for a dual‐panel prostate‐dedicated PET system using both simulated and experimental studies. Quantitative and qualitative results showed that deep learning‐based post‐processing achieved the highest lesion contrast and image consistency, particularly for small and low‐contrast regions. While regularized and PSF‐based methods improved specific aspects of image quality, the Swin‐based approach consistently outperformed conventional techniques without increasing background noise. These findings demonstrate the potential of combining physical modeling with learned enhancement to improve image quality in limited‐angle PET scanners, supporting its use in high‐resolution prostate‐targeted imaging. The current automated 22‐step acquisition framework could also support future extensions toward scanner quality‐assurance procedures.

## CONFLICT OF INTEREST STATEMENT

The authors declare that they have no known competing financial interests or personal relationships that could have appeared to influence the work reported in this paper.

## Supporting information



Supporting Information

## Data Availability

The simulation data used in this work are available from the authors.
