# Supplementary material for: Image reconstruction and elongation artifact reduction for a dual‐panel dedicated prostate PET scanner
Source: Med Phys. 2026 Jan 27;53(2):e70298. doi: 10.1002/mp.70298 (PMC12836138; doi:10.1002/mp.70298)
Supplement: Supplementary file 1 — Supporting Information [file MP-53-0-s001.pdf]

Supplementary Material

Supplementary Table 1. Coordinate positions (in mm) for the 22-step dual-panel acquisition protocol of the Provision PET system. Each row lists the X and Z translations of the upper and lower detector panels for the corresponding acquisition step, defining the scanner's motion range used in the study.

ID	Step Name	UpperX (mm)	UpperZ (mm)	LowerX (mm)	LowerZ (mm)
1	Step 1	0	0	0	0
2	Step 2	0.6	0	0.6	0
3	Step 3	46.8	0	0	0
4	Step 4	-46.8	0	0	0
5	Step 5	0	0	46.8	0
6	Step 6	0	0	-46.8	0
7	Step 7	23.4	9	23.4	9
8	Step 8	23.4	-9	23.4	-9
9	Step 9	-23.4	9	-23.4	9
10	Step 10	-23.4	-9	-23.4	-9
11	Step 11	46.8	0	-46.8	0
12	Step 12	-46.8	0	46.8	0
13	Step 13	57.2	0	-104	0
14	Step 14	-57.2	0	104	0
15	Step 15	104	0	-57.2	0
16	Step 16	-104	0	57.2	0
17	Step 17	104	0	-104	0
18	Step 18	-104	0	104	0
19	Step 19	0	22.5	0	-22.5
20	Step 20	0	-22.5	0	22.5
21	Step 21	0	22.5	0	22.5
22	Step 22	0	-22.5	0	-22.5

Supplementary S1 – Shape-Sensitive Loss Function

The SWIN network was trained using a composite loss that balances overall similarity, edge alignment, and intensity fidelity between the predicted and reference PET images. The total loss is defined as

$$\mathcal{L}_{\text{total}}(t) = \alpha(t)\,\mathcal{L}_{\text{SSIM}} + \beta(t)\,\mathcal{L}_{\text{edge}} + \gamma(t)\,\mathcal{L}_{\text{Huber}}$$

Components:

$$\begin{aligned}
\mathcal{L}_{\text{SSIM}} &= 1 - \text{SSIM}(\hat{y}, y),\\
\mathcal{L}_{\text{edge}} &= \frac{1}{3}(\| \nabla_x \hat{y} - \nabla_x y \|_1 + \| \nabla_y \hat{y} - \nabla_y y \|_1 + \| \nabla_z \hat{y} - \nabla_z y \|_1),\\
\mathcal{L}_{\text{Huber}} &= \begin{cases} \frac{1}{2}(\hat{y} - y)^2 \quad , & | \hat{y} - y | < \delta, \\ \delta(| \hat{y} - y | - \frac{1}{2}\delta), & \text{otherwise,} \end{cases}
\end{aligned}$$

where $\delta = 0.1$.

These terms respectively measure structural similarity, edge consistency, and smooth intensity difference.

Dynamic weighting:

The weights $\alpha(t)$, $\beta(t)$, and $\gamma(t)$are smoothly varied during training to balance global and local detail recovery:

$$\begin{aligned}
\alpha(t) &= \alpha_{\text{min}} + \frac{1}{2}(\alpha_{\text{max}} - \alpha_{\text{min}})(1 + \cos(\pi t/T)),\\
\gamma(t) &= \gamma_{\text{min}} + \frac{1}{2}(\gamma_{\text{max}} - \gamma_{\text{min}})(1 - \cos(\pi t/T)),\\
\beta(t) & \quad \text{remains constant and small.}
\end{aligned}$$

This schedule gradually shifts the emphasis from global structure (SSIM) to edge refinement (edge + Huber) as training proceeds. Exact parameter ranges follow standard cosine-annealing practice and were chosen for stable convergence.
